# Human bone marrow-derived mesenchymal stem overexpressing microRNA-124-3p inhibit DLBCL progression by downregulating the NFATc1/cMYC pathway

**DOI:** 10.1186/s13287-023-03373-w

**Published:** 2023-05-29

**Authors:** Xiaoxuan Zhao, Mingxi Xu, Xuemeng Hu, Xiaolei Ding, Xian Zhang, Liye Xu, Li Li, Xiuhua Sun, Jincheng Song

**Affiliations:** 1 Department of Oncology, The Second Affiliated Hospital of Dalian Medical University, Dalian, 116023 Liaoning People’s Republic of China; 2 Department of Dermatology, Dalian Dermatosis Hospital, Dalian, 116021 Liaoning People’s Republic of China; 3 Rheumatology Department, The Second Affiliated Hospital of Dalian Medical University, Dalian, 116023 Liaoning People’s Republic of China; 4 Department of Hematology, The Second Affiliated Hospital of Dalian Medical University, Dalian, 116023 Liaoning People’s Republic of China; 5 Graduate School of Dalian Medical University, Dalian, People’s Republic of China; 6 Graduate School of China Medical University, Shenyang, People’s Republic of China

**Keywords:** Human bone marrow mesenchymal stem cells, DLBCL, Exosomes, MicroRNA-124-3p, NFATc1

## Abstract

**Background:**

Exosomes play important roles in intercellular communication by delivering microRNAs (miRNAs) that mediate tumor initiation and development, including those in diffuse large B cell lymphoma (DLBCL). To date, however, limited studies on the inhibitory effect of exosomes derived from human bone marrow mesenchymal stem cells (hBMSCs) on DLBCL progression have been reported. Therefore, this study aimed to investigate the role of hBMSC exosomes carrying microRNA-124-3p in the development of DLBCL.

**Methods:**

Microarray-based expression analysis was adopted to identify differentially expressed genes and regulatory miRNAs, which revealed the candidate NFATc1. Next, the binding affinity between miR-124-3p and NFATc1 was detected by luciferase activity assays. The mechanism underlying NFATc1 regulation was investigated using lentiviral transfections. Subsequently, DLBCL cells were cocultured with exosomes derived from hBMSCs transfected with a miR-124-3p mimic or control. Proliferation and apoptosis were measured in vitro. Finally, the effects of hBMSC-miR-124-3p on tumor growth were investigated in vivo.

**Results:**

MiR-124-3p was expressed at low levels, while NFATc1 was highly expressed in DLBCL cells. MiR-124-3p specifically targeted and negatively regulated the expression of NFATc1 in DLBCL cells, upregulated miR-124-3p-inhibited DLBCL cell proliferation and promoted apoptosis. The miR-124-3p derived from hBMSCs inhibits tumor growth both in vivo and in vitro via downregulation of the NFATc1/cMYC pathway.

**Conclusion:**

Human bone marrow-derived mesenchymal stem cell overexpressing microRNA-124-3p represses the development of DLBCL through the downregulation of NFATc1.

**Supplementary Information:**

The online version contains supplementary material available at 10.1186/s13287-023-03373-w.

## Introduction

Diffuse large B cell lymphoma is a malignant tumor derived from B cells. The 5-year survival rate of late-stage DLBCL patients is less than 30% [1]. Although the cure rate has been improved with the emergence of new treatment methods, such as CAR-T therapy, more than 50% of patients still experience relapse/refractory disease [2, 3]. Therefore, effective new treatment approaches that can prolong DLBCL patient survival are urgently needed.

Recently, an increasing number of studies has proposed the beneficial application of bone marrow-derived mesenchymal stem cells (BMSCs) as a potential approach for treating malignant tumors [4]. hBMSCs can secrete a large number of exosomes, with characteristics of good histocompatibility, high efficiency of internal delivery, and ease of collection [5]. Accumulating evidence indicates that exosomes play a crucial role in modulating intercellular communication and serve as important mediators in DLBCL [6, 7]. Exosomes secreted by hBMSCs can deliver intracellular contents, such as proteins, messenger RNAs (mRNAs), and microRNAs (miRNAs), to recipient cells [8]. Because miRNA is relatively stable in exosomes, it is promising to further explore a novel therapeutic method based on hBMSC-derived exosome-encapsulated miRNA. MiR-124-3p has been shown to act as a tumor suppressor in several malignancies, including breast [9], cervical [10] and colorectal cancer [11]. NFATc1, as a transcription factor, can promote the growth of lymphoma [12]. In the present study, we found that miR-124-3p negatively regulates nuclear factor of activated T cells c1 (NFATc1). We therefore aimed to explore the therapeutic effect of miR-124-3p derived from hBMSCs on DLBCL by regulating NFATc1 to provide potential therapeutic strategies.

## Materials and methods

### Microarray-based expression analysis and GEO database analysis

The DLBCL-related miRNA expression profiles of GSE29493 and GSE40239 were retrieved from the Gene Expression Omnibus (GEO) database (https://www.ncbi.nlm.nih.gov/geo/).

MiR-124’s pan cancer analysis is conducted through online websites (http://www.picb.ac.cn/dbDEMC/).

### Bioinformatics predicted NFATc1 as the target of miR-124-3p

The miRbase (http://www.mirbase.org/), miRDB (http://mirdb.org), and TargetScan (http://www.targetscan.org) databases were employed to predict the genes that might be regulated by miR-124-3p [13]. The prognostic genes of DLBCL were obtained from the GEO (GSE10846) database [14]. The differentially expressed genes in DLBCL were downloaded from the GEPIA website (http://gepia.cancer-pku.cn/) [15]. We intersected the miR-124-3p-related genes and the differentially expressed genes and the prognostic genes in DLBCL. Subsequently, genes were subjected to Kyoto Encyclopedia of Genes and Genomes (KEGG) pathway analysis [16], Gene Ontology (GO) analyses [17], and protein–protein interaction (PPI) analysis [18]. The network was analyzed by Cytoscape 3.7.2 software. Then, we used the microRNA database (http://www.microrna.org/) to predict the miR-124-3p binding site on NFATc1 mRNA. The expression of NFATc1 from The Cancer Genome Atlas (TCGA) was analyzed with datasets from the GEPIA and Oncomine (www.oncomine.org) databases. Transcription factors were predicted through the hTFtarget website (http://bioinfo.life.hust.edu.cn).

### Cell cultures

Human SU-DHL-6 and SU-DHL-10 cells were a kind gift from Professors Jing Wei and Fang Wang of the Biology Laboratory of Sichuan University (Sichuan, China). The cell lines were authenticated by short tandem repeat analysis. The cells were cultured in 1640 medium (HyClone, Logan, USA) supplemented with 10% fetal bovine serum (HyClone), 100 units/mL penicillin and 100 µg/mL streptomycin and were maintained in a humidified atmosphere at 37 °C with 5% CO_2_.

### hBMSC identification and culture

hBMSCs were purchased from Saiye Biology Co., Ltd. (HUXMA-01001) and cultured in DMEM-F12 culture medium with 10% FBS at 37 °C with 5% CO_2_. hBMSCs were subjected to osteogenic and adipogenic differentiation in OriCell™ medium (Cyagen Biosciences Inc., Guangzhou, China) and then stained with alizarin red or oil red O according to the manufacturer’s instructions. A flow cytometer (BD Biosciences Pharmingen, San Jose, CA, USA) was used to detect the expression of hBMSC surface markers.

### Transfection and lentiviral transduction

DLBCL cells were transfected with miR-124-3p/negative control (NC) mimic, miR-124-3p/NC inhibitor, or sh-NFATc1/NC plasmids (GenePharma, Shanghai, China) using RFectSPsRNA Transfection reagent (Baidai, Shanghai, China) in accordance with the manufacturer’s instructions for 48 h. Lentivirus packaging was performed in cells seeded in 60-mm dishes and transfected with pMD2G, psPAX2 and pLENTI 6.3-Luciferase/miR-124-3p (miR-NC/miR-124-3p) (4 μg) plasmids. Twenty-four hours after transfection, the medium was replaced with fresh medium, and the cells were cultured for another 24 h. Then, the medium was collected and added to target cells for infection purposes. hBMSCs were cultured in 24-well plates at a density of 5 × 10^4^ cells/well overnight before infection. Five hundred microliters of lentivirus-containing medium and 500 µL of fresh culture medium supplemented with 8 µg of polyacrylamide (Sigma, St. Louis, MO, USA) was added to each well. The plates were then centrifuged at 3000 × g at 37 °C for 1 h before the virus-containing medium was replaced with fresh medium.

### Dual luciferase reporter assay

The artificially synthesized NFATc1 3′-untranslated region (3′-UTR) gene fragment was cloned into the psiCHECK-2 dual luciferase vector to construct psiCHECK-2-NFATc1-3′-UTR-wild-type (NFATc1-WT) and psiCHECK-2-NFATc1-3′-UTR-mutant (NFATc1-MUT) vectors. The WT and MUT plasmids were cotransfected with the miR-124-3p mimic and NC mimic. The cells were collected and lysed after 24 h of transfection. Luciferase activity was detected using a Dual Luciferase Reporter Kit (Promega, Madison, WI, USA).

### Exosome isolation

Exosomes were isolated from the serum using an ExoQuick Kit (EXOQ20A-1, System Biosciences, Palo Alto, CA, USA) in strict accordance with the instructions provided by the manufacturer. Ultracentrifugation was used for the isolation of exosomes from cultured hBMSCs [19]. The exosomes were resuspended in 100 μL of PBS and stored at -80 °C. The content of exosomes was determined by the BCA method.

### Observation of exosomes under TEM and analysis with NTA

Transmission electron microscopy (TEM) was used to identify exosomes. Samples were examined under an electron microscope (HITACHI, Tokyo, Japan) after drying at an accelerating voltage of 80 kV. To determine the size distribution, exosomes were analyzed with a Zeta View PMX110 nanoparticle tracking analyzer (Particle Metrix, Germany). DiO is used to track exosomes, as it can stably bind to the lipid region of the exosome membrane. All experimental steps were performed following the instructions of the DiO-Membrane EVS Labeling and Purification Kit (Rengen Biosciences Co., Ltd., Liaoning, China). The uptake of exosomes by DLBCL cells was observed under a fluorescence microscope (FV3000, Olympus, Tokyo, Japan).

### Coculture and inhibition of exosome secretion

The exosomes produced by hBMSCs transfected with either miR-124-3p mimic or NC mimic were added to DLBCL cells cultured in exosome-free medium in a 6-well plate (Fig. 6A); the cells were categorized as Exo-miR-NC + DLBCL and Exo-miR-124-3p + DLBCL. MiR-124-3p-transfected hBMSCs (5 × 10^4^) and DLBCL cells (1 × 10^5^) were cocultured in a Transwell® model (Fig. 7A). hBMSCs and DLBCL cells were cocultured for 72 h before they were collected and used for subsequent experiments. The specific inhibitor GW4869 (Sigma-Aldrich, St. Louis, MO, USA) and DMA (Santa Cruz, Paso Robles, CA, USA) were used to block exosome secretion at concentrations of 10 nM and 15 nM, respectively.

### Reverse transcription quantitative polymerase chain reaction (RT-qPCR)

qPCR was performed according to the instructions of the RT-qPCR Kit (Fermentas Inc., Hanover, MD, USA) with primers synthesized by TaKaRa (Tokyo, Japan) (shown in Additional file 1: Table S1). Real-time quantitative PCR (qPCR) was performed by a quantitative PCR instrument (Bio-Rad iQ5, Bio-Rad, Richmond, CA, USA).

### Western blot analysis and immunohistochemistry were performed as previously described

The protein was quantified by a bicinchoninic acid (BCA) kit (Thermo Fisher Scientific, Rockford, IL, USA). Primary antibodies against CD63 (1:1000, Abcam, UK), Hsp70 (1:1000, Abcam, UK), calnexin (1:1000, Abcam, UK), TSG 101 (1:1000, Abcam, UK), cMYC (1:1000, Cell Signaling Technology Danvers, MA, USA), and GAPDH (1:5000, Abcam, UK) and mouse antibodies against NFATc1 (1:1000, Santa Cruz, CA, USA) were used. Immunohistochemical staining was performed using the streptavidin-peroxidase method.

### Apoptosis assay and cell proliferation assay

Apoptosis was assessed by standard annexin V-FITC and/propidium iodide (PI) double staining analysis on a flow cytometer. Apoptosis was analyzed using a BD FACSCanto II flow cytometer (BD Biosciences, CA, USA) at 488 nm. Data were analyzed by FlowJo software. The cell proliferation assay was performed by CCK-8. The absorbance at 450 nm was recorded on a microplate reader (Bio-Rad Laboratories, Hercules, CA, USA).

### Tumor xenografts in nude mice

NOD/SCID mice are used in our experiment because of their low immunity and easy tumorigenicity. A total of 12 male NOD/SCID mice (4–6 weeks old, weighing 18–22 g) were purchased from Huafukang Biotechnology Co., Ltd. (Beijing, China). After one week of acclimation, 100 μL SU-DHL-10 cells (1 × 10^7^) were subcutaneously injected into the upper flank region. Tumor volumes were measured every 2 days according to the formula *V* = 1/2 * (short diameter)^2^ × (longest diameter). After the tumor reached an approximate volume of 100 mm^3^, the mice were randomly (draw by lot) divided into three groups (hBMSC-transfected miR-124-3p, hBMSC-transfected miR-NC and PBS; four animals per group) by my colleagues. hBMSCs transfected with miR-124-3p or miR-NC were injected into NOD/SCID mice via the tail vein once every three days (5 × 10^5^ cells/mouse); mice in the PBS group received an equivalent volume of PBS. After seven injections, the mice were euthanized by cervical dislocation after CO2 anesthesia, and tumors were removed and weighed. Finally, the tumor samples were frozen in liquid nitrogen or embedded in paraffin for immunohistochemistry analysis after imaging. This study was carried out in compliance with the ARRIVE guidelines.

### Statistical analysis

Statistical analyses were performed with SPSS 21.0 statistical software (SPSS, Inc., Chicago, IL, USA). Comparisons between two groups were performed with an independent samples *t test*; comparisons among multiple groups were performed with one-way analysis of variance (ANOVA). A statistically significant difference was defined as *P* < 0.05. Data are presented as the means ± SD of three independent experiments (*P < 0.05, ** P < 0.01, *** P < 0.001). Drawing through GraphPad Prism version 6 (GraphPad Prism Inc., San Diego, CA).

## Results

### Downregulation of miR-124-3p in DLBCL

**Fig. 1:**
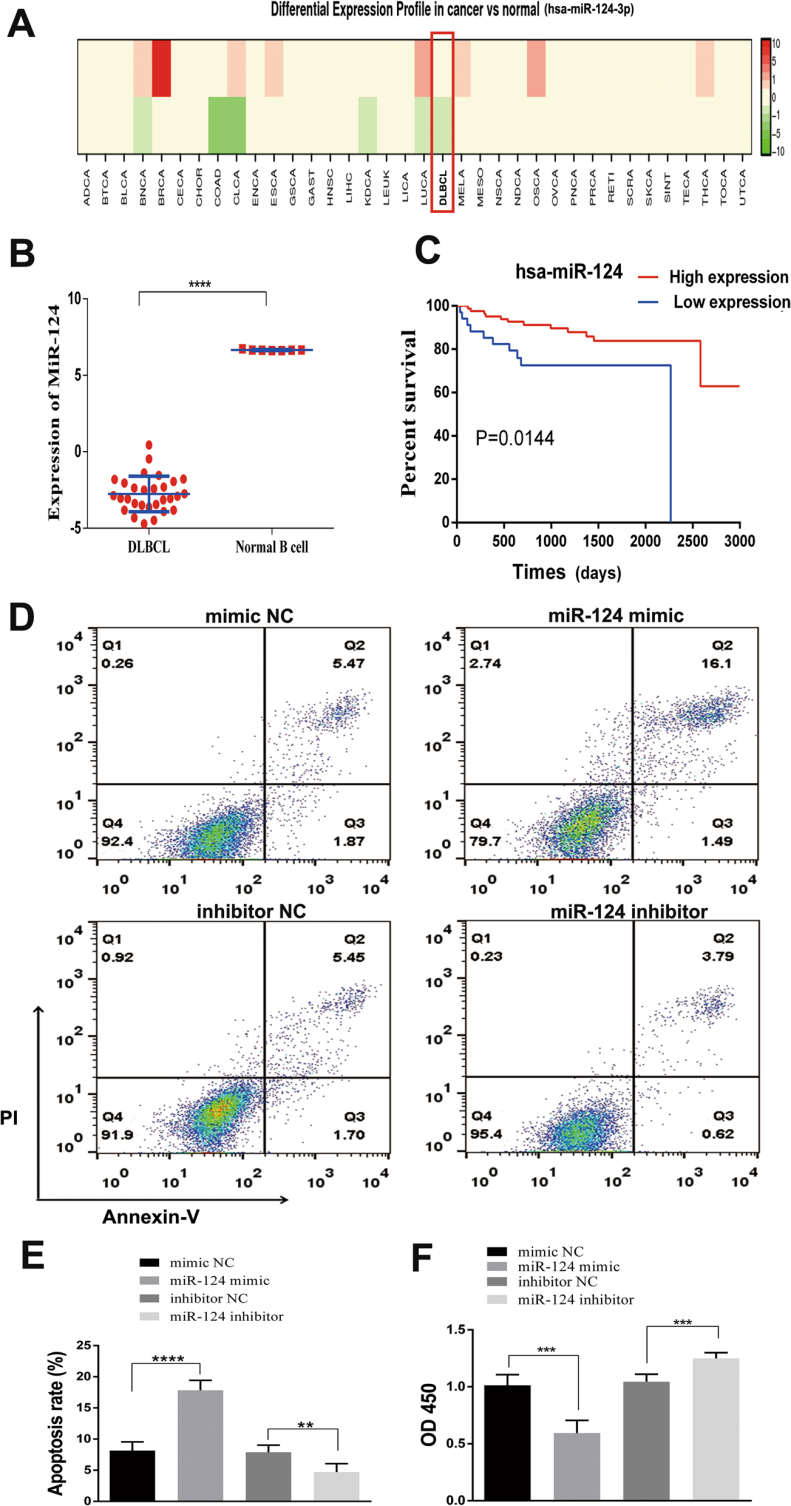
Low miR-124-3p expression in DLBCL. **A** Differential expression profile of miR-124-3p in different cancers and normal tissues (GSE2399). **B** The expression of miR-124-3p is lower in DLBCL cells than in normal B cells (GSE29493). **C** Low miR-124-3p expression levels in DLBCL indicate a worse prognosis and shorter overall survival (OS) than high miR-124-3p expression levels (GSE40239). **D** Overexpression of miR-124-3p promotes apoptosis in DLBCL cells. **E** Quantification of figure D. **F** Upregulated miR-124-3p expression inhibits DLBCL cell proliferation. Data are presented as the means ± SD of three independent experiments (**P < 0.01; ***P < 0.001; ****P < 0.0001)

The microRNA-124-3p (miR-124-3p) has been reported to have a tumor suppressive effect in many tumors [20]. The DLBCL-related microarray (GSE2399 and GSE29493) indicated that miR-124-3p expression was lower in DLBCL cells than in normal B cells (Fig. 1A, B). Furthermore, we observed that low miR-124-3p expression levels in DLBCL cells indicated a shorter overall survival (OS) than high miR-124-3p expression levels (GSE40239) (Fig. 1C).

### Overexpression of miR-124-3p inhibits proliferation and promotes apoptosis in DLBCL cells

To investigate the biological function of miR-124-3p, DLBCL cells were treated with a mimic and an inhibitor of miR-124-3p. The apoptosis rate was elevated in the miR-124-3p mimic group compared with the mimic-NC group (*P* < 0.05) (Fig. 1D, E). When miR-124-3p was inhibited, cell proliferation was increased (Fig. 1F).

### Bioinformatics analysis predicted NFATc1 as a target of miR-124-3p

To further predict the target genes regulated by miR-124-3p, three miRNA-mRNA relation prediction databases (TargetScan, miRTarBase, and mirRDB) were used, and a total of 1027 genes were identified as miR-124-3p-related genes (Additional file 2: Fig. S1A). After intersecting the differentially expressed genes (GEPIA website) and prognostic genes (GSE10846), 237 genes were obtained (Additional file 2: Fig. S1B). These genes are displayed in Additional file 2: Fig. S1D. The genes were then subjected to GO, PPI network, and KEGG pathway analyses. GO functional enrichment analysis revealed that biological regulation, metabolic process, and response to stimulus were the main biological processes. Concerning cellular components, the major components of these genes include the membrane, nucleus, and membrane-enclosed lumen. These genes are related to various molecular functions, including protein binding, ion binding, and nucleic acid binding (Additional file 2: Fig. S1C). PPI analysis showed that NFATc1 was one of the hub genes (Additional file 2: Fig. S1E). Further KEGG enrichment analysis showed that these genes were mainly concentrated in the signaling pathways “blood vessel development,” “regulation of cell adhesion” and “positive regulation of cell cycle” (Additional file 2: Fig. S1F).

### MiR-124-3p targets and downregulates NFATc1

**Fig. 2:**
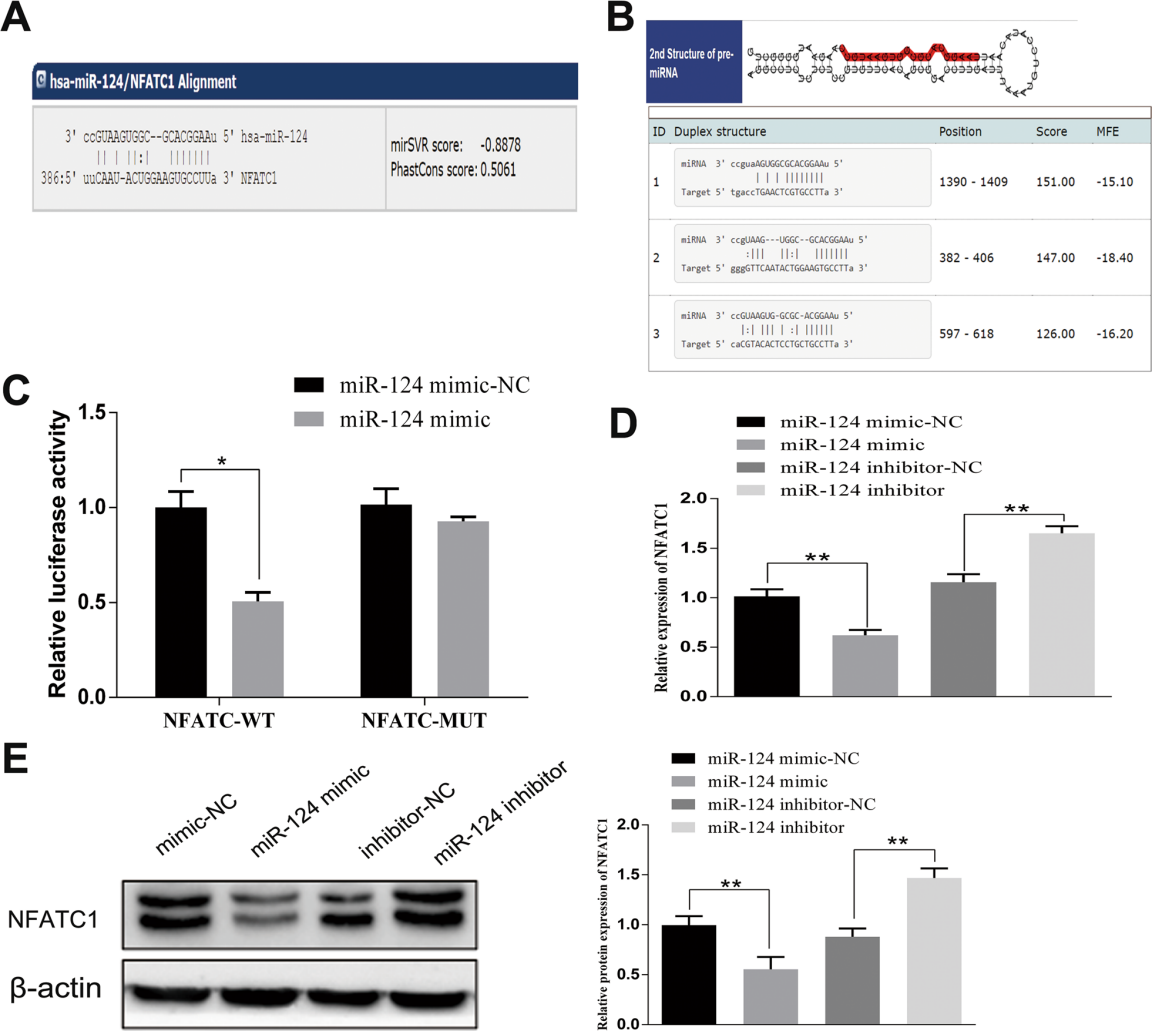
MiR-124-3p targets and negatively regulates NFATc1. The targeted binding site between NFATc1 and miR-124-3p was predicted through the microRNA database (**A**) and TargetScan database (**B**). **C** The interaction between miR-124-3p and NFATc1 was confirmed by a dual luciferase reporter gene assay. NFATc1 expression was detected by RT-PCR (**D**) and western blot (**E**). Human SU-DHL-10 cells were used in our experiments (authenticated by short tandem repeat analysis). The data are expressed as the means ± SD; comparisons between two groups were analyzed using unpaired t tests, and the experiment was repeated three times independently (*P < 0.05; **P < 0.01)

The miR-124-3p binding site on NFATc1 was predicted through the microRNA database (http://www.microrna.org/) (Fig. 2A) and TargetScan (Fig. 2B). The dual luciferase reporter gene assay results showed that compared to the NC group, the NFATc1 WT 3′-untranslated region (UTR) group showed significant inhibition of luciferase activity by miR-124-3p (*P* < 0.05), while no difference was observed in the NFATc1 3′-UTR MUT group (*P* > 0.05) (Fig. 2C). To further verify the effect of miR-124-3p on NFATc1, qRT-PCR (Fig. 2D) and western blot analysis (Fig. 2E) were employed to determine the mRNA and protein expression, respectively, of NFATc1. NFATc1 expression at both the mRNA and protein levels was decreased in the miR-124-3p mimic group, whereas this trend was reversed when miR-124-3p was inhibited. These findings indicate that NFATc1 is a target gene of miR-124-3p and that miR-124-3p negatively regulates NFATc1.

### Silencing NFATc1 inhibits proliferation and promotes apoptosis in DLBCL cells

**Fig. 3:**
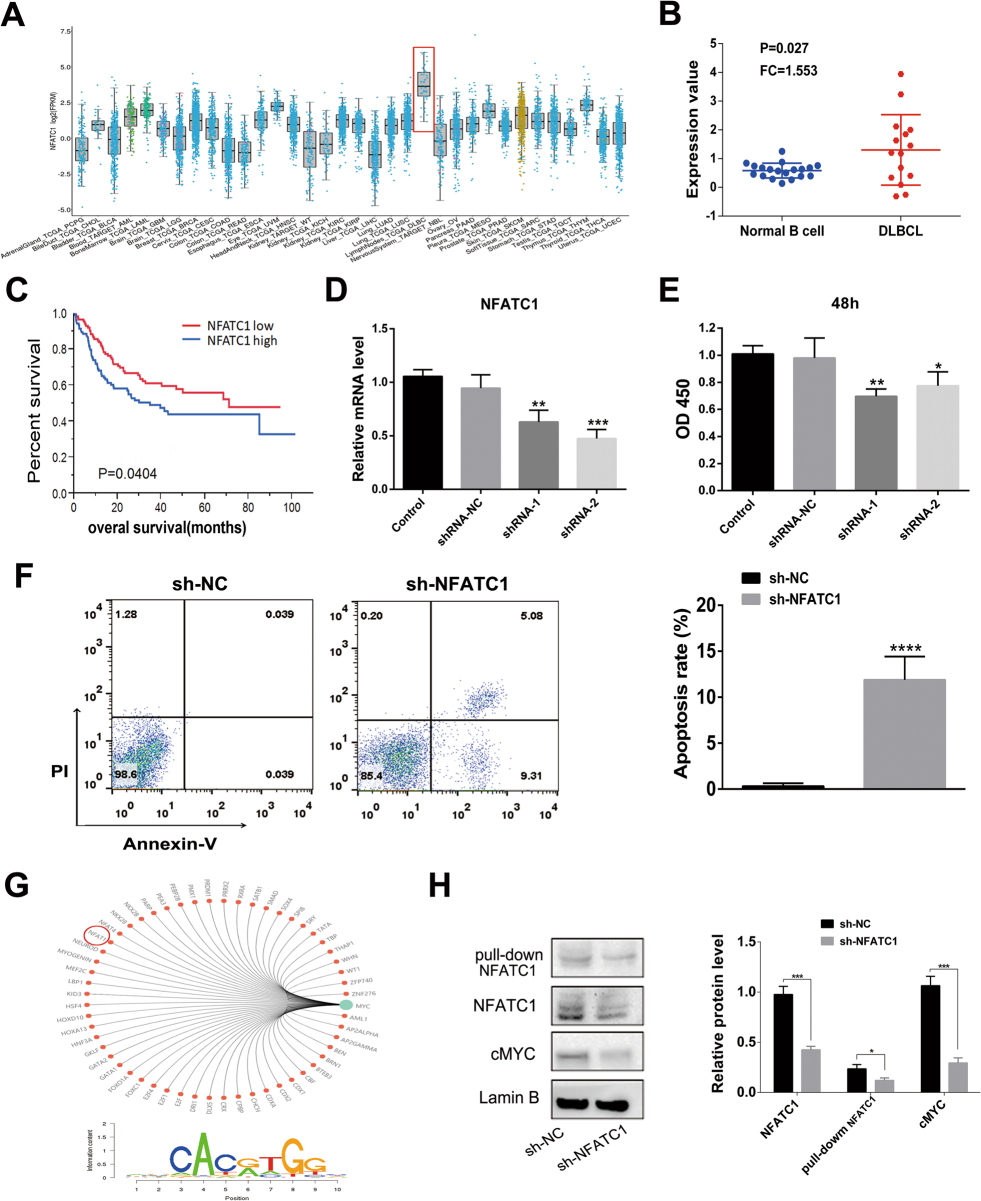
Silencing NFATc1 inhibits proliferation and promotes apoptosis in DLBCL cells. **A** The expression profile of NFATc1 in different cancers (TCGA database). **B** NFATc1 is highly expressed in DLBCL (Oncomine dataset). **C** Patients with high NFATc1 expression have a poor prognosis. **D** The knockdown efficiency of sh-NFATc1 was evaluated by RT-PCR. **E** Proliferation was suppressed when NFATc1 was knocked down. **F** Knocking down NFATc1 promoted apoptosis. **G** cMYC was one of the NFATc1-regulated genes predicted through the hTFtarget website, and the binding sites are displayed. **H** NFATc1 and cMYC protein expression in cells after knocking down NFATc1 determined by western blot analysis. A pulldown assay was performed to analyze the binding of the NFATc1 protein to the cMYC promoter. Data are presented as the means ± SD of three independent experiments (*P < 0.05; ***P < 0.001; ****P < 0.0001)

NFATc1 is highly expressed in DLBCL (TCGA database, Fig. 3A) (Oncomine database, Fig. 3B) and is related to a poor prognosis (Fig. 3C) in DLBCL. To explore the effect of NFATc1 on DLBCL cells, the NFATc1 gene was silenced with sh-NFATc1, and the knockdown efficiency was evaluated by RT-PCR analysis (Fig. 3D). Silencing NFATc1 obviously suppressed cell proliferation (Fig. 3E) and promoted apoptosis (Fig. 3F). As a transcription factor, cMYC was one of the genes regulated by NFATc1 (hTFtarget website), and the binding sites are displayed in Fig. 3G. There was a significant decline in cMYC and NFATc1 expression in NFATc1-silenced cells (Fig. 3H).

### Overexpression of NFATc1 overcomes the biological function of miR-124-3p

**Fig. 4:**
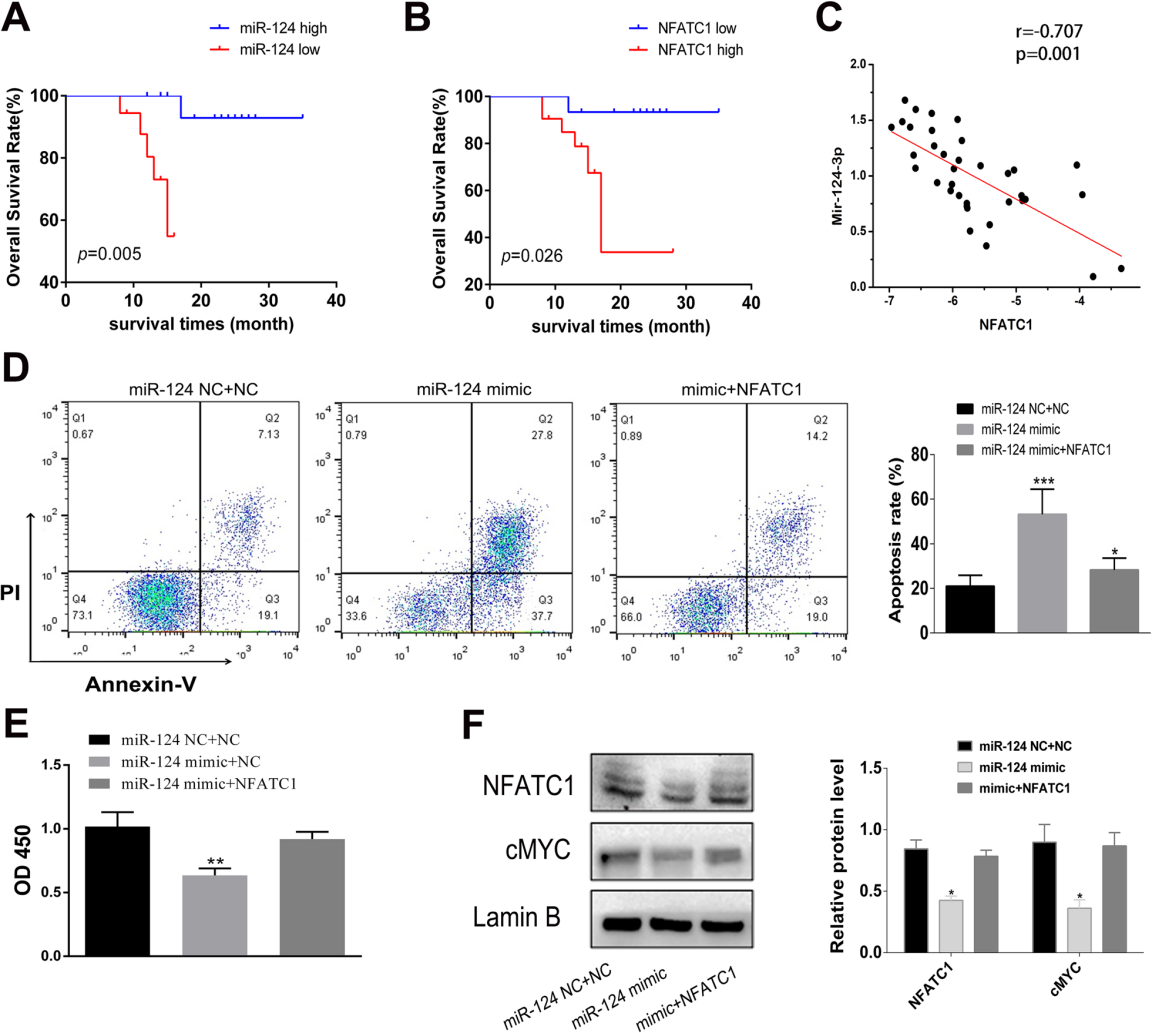
The biological function of miR-124-3p was reversed by ectopic expression of NFATc1. **A** Our data suggests that high miR-124-3p expression is associated with a good prognosis. **B** High NFATc1 expression indicates a poor prognosis based on our own data. **C** A negative correlation between miR-124-3p and NFATc1 expression was observed in 36 DLBCL tissues. **D** The increased apoptosis observed by miR-124-3p overexpression was reversed by cotransfection with an NFATc1 overexpression vector. **E** The inhibitory effect of miR-124-3p upregulation on DLBCL cell proliferation was significantly reversed by NFATc1 overexpression. **F** NFATc1 and cMYC protein expression was inhibited by miR-124-3p overexpression and reversed by cotransfection with a NFATc1 overexpression vector. Data are presented as the means ± SD of three independent experiments (*P < 0.05; ***P < 0.001)

To investigate the relevance of miR-124-3p and NFATc1 in human DLBCL, we performed PCR to determine the expression of miR-124-3p and NFATc1 in 36 primary DLBCL samples. There was a significant correlation of the miR-124-3p level with stage, IPI score, and extranodal invasion. The NFATc1 level was also correlated with stage and IPI score but not with other clinical features (Table 1). The Kaplan–Meier survival curve showed that increased miR-124-3p expression and low NFATc1 expression were related to good survival (Fig. 4A, B). A negative correlation between miR-124-3p and NFATc1 was observed in the 36 DLBCL tissues (*r* =  − 0.707, *P* = 0.001, Fig. 4C). Furthermore, the miR-124-3p mimic was cotransfected with the NFATc1 overexpression plasmid. Exogenous overexpression of NFATc1 overcame the effects of miR-124-3p on DLBCL cells (Fig. 4D, E). NFATc1 and cMYC protein expression was inhibited by miR-124-3p and reversed by cotransfection with the NFATc1 overexpression plasmid (Fig. 4F).

**Table 1:** Correlation between the clinicopathological characteristics and expression of miR-124 and NFATc1 in DLBCL patients

Variables	No. (%)	miR-124 expression (Mean ± SEM)	*P* value	NFATC1 expression (Mean ± SEM)	*P* value
Age (years)			0.239		0.863
< 60	11 (30.5)	− 5.14 ± 0.31		0.98 ± 0.11	
≥ 60	25 (69.5)	− 5.80 ± 0.16		1.0 ± 0.08	
Gender			0.126		0.064
Male	20 (55.5)	− 5.88 ± 0.16		1.10 ± 0.07	
Female	16 (44.5)	− 5.42 ± 0.26		0.86 ± 0.10	
IPI score			**0.046**		**0.035**
0–2	14 (38.9)	− 5.31 ± 0.25		0.83 ± 0.11	
3–5	22 (61.1)	− 5.91 ± 0.17		1.10 ± 0.07	
Stage			**0.030**		0.110
I + II	20 (55.5)	− 5.40 ± 0.22		0.90 ± 0.09	
III + IV	16 (44.5)	− 6.0 ± 0.17		1.11 ± 0.09	
ESR			0.185		0.456
Normal	11 (30.5)	− 5.32 ± 0.35		0.93 ± 0.14	
High	25 (69.5)	− 5.84 ± 0.14		1.03 ± 0.07	
Extranodal invasion			**0.031**		**0.004**
1 site	23 (19.8)	− 5.91 ± 0.15		1.13 ± 0.06	
> 1 site	13 (80.2)	− 5.26 ± 0.28		0.76 ± 0.11	
LDH			0.138		0.249
Low	18 (50)	− 5.45 ± 0.22		0.92 ± 0.09	
High	18 (50)	− 5.90 ± 0.19		1.07 ± 0.09	
Microglobulin			0.763		0.526
Low	23 (80.2)	− 5.71 ± 0.16		1.03 ± 0.08	
High	13 (19.8)	− 5.61 ± 0.30		0.94 ± 0.11	
B symptom			0.109		0.121
Yes	22 (61.1)	− 5.86 ± 0.15		1.09 ± 0.06	
No	14 (38.9)	− 5.38 ± 0.29		0.86 ± 0.13	

The significance of bold means that the results are significantly different

*ESR* Erythrocyte sedimentation rate, *LDH* Lactate dehydrogenase, *NFATc1* Nuclear factor of activated T cells c1, *DLBCL* Diffuse large B cell lymphoma

We further studied the significance of miR-124-3p and NFATc1 in cell proliferation/apoptosis by overexpressing NFATc1 with wild-type and mutated 3′-UTR ± miR-124-3p. As Additional file 3: Fig. S2 shows, the survival rate of cells decreased significantly in the WT-NFATc1 + miR-124-3p group compared with the WT-NFATc1 + miR-124-NC group and increased in the MUT-NFATc1 + miR-124-3p group (Additional file 3: Fig. S2A). In the apoptosis experiment, the apoptosis rate of cells increased in the miR-124-3p mimic groups, and the apoptosis rate increased the most when the 3′-UTR of NFATc1 was mutated (Additional file 3: Fig. S2B).

### Isolation and identification of hBMSCs

After three to four passages, we observed a relatively large number of purified hBMSCs with a shuttle shape and swirling arrangement (Additional file 4: Fig. S3A). The ability of hBMSCs to differentiate was assessed. Oil red O staining confirmed that the hBMSCs could undergo adipogenic differentiation (Additional file 4: Fig. S3B). After 4 weeks of osteogenic differentiation, many red calcium nodules were observed at the cell center based on alizarin red staining (Additional file 4: Fig. S3C). Antibodies against CD105, CD90, CD31, CD34, CD45, CD166, CD29, CD11b, HLA-DR, and CD73 were used to identify the cell type by flow cytometry. We found that CD29 (99.74%), CD90 (99.81%), CD105 (99.32%), CD166 (96.33%), and CD73 (99.73%) were expressed, while CD34, CD31, CD45, CD11b, and HLA-DR were not. These results support that the cultured cells were hBMSCs (Additional file 4: Fig. S3D).

### Collection of exosomes secreted from hBMSCs

TEM was used to identify BMSC-derived exosomes. The exosomes exhibited a globular or oval shape and presented a complete lipid membrane (Additional file 4: Fig. S3E). The Zeta View nanoparticle tracking analyzer revealed that most of the exosome particles were approximately 100 nm in size (Additional file 4: Fig. S3F). Western blot analysis showed that the exosome surface marker proteins TSG 101, CD63, and Hsp70 were expressed in BMSC-derived exosomes, whereas calnexin was not (Additional file 4: Fig. S3G). The miR-124-3p level in miR-124-3p-transfected hBMSCs and exosomes was significantly higher than that in the respective control (Additional file 4: Fig. S3H).

### hBMSCs deliver miR-124-3p to DLBCL cells by secreting exosomes

**Fig. 5:**
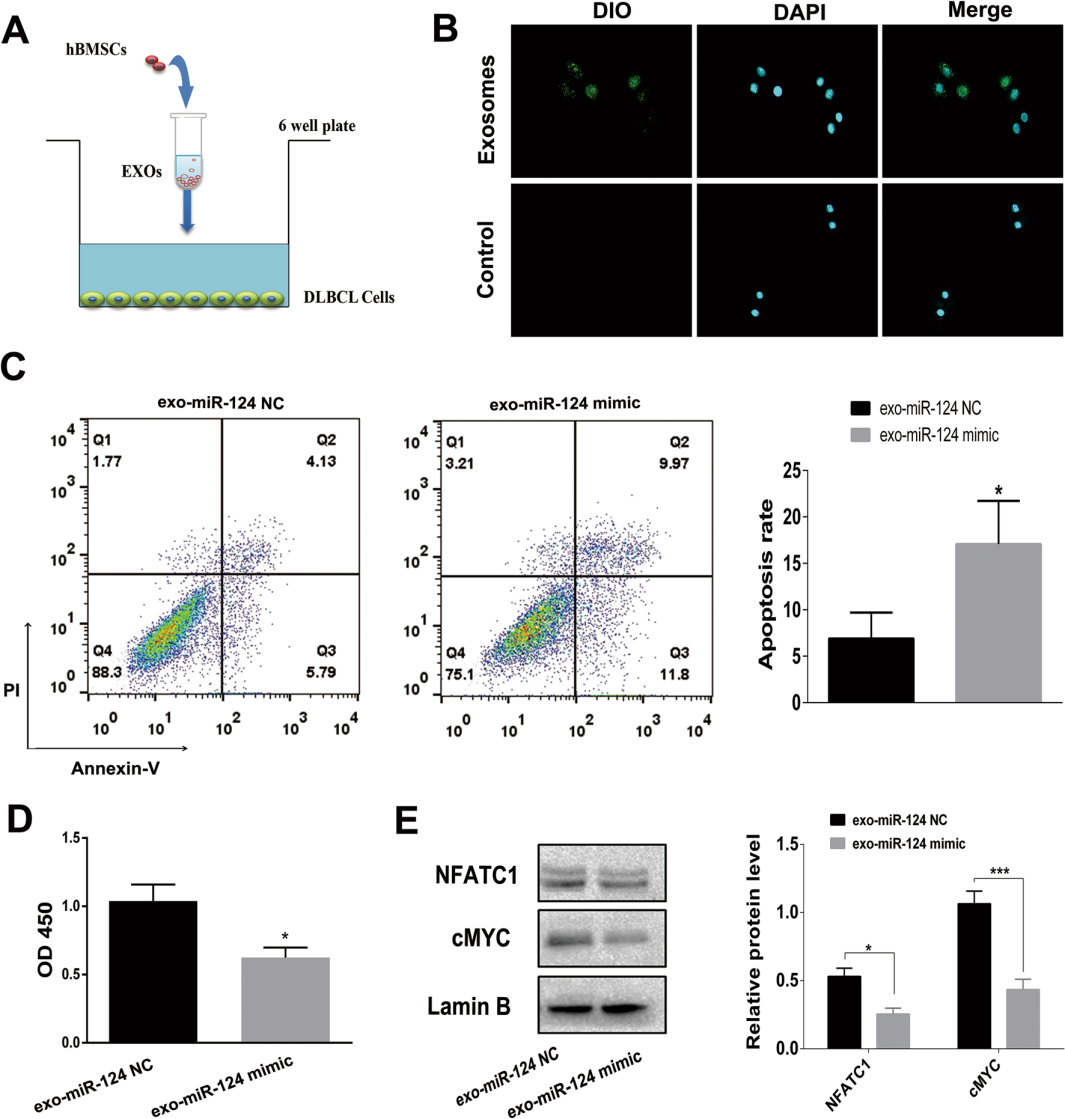
hBMSC-derived exosomal miR-124-3p suppresses DLBCL cell proliferation and enhances apoptosis. **A** Schematic illustrating the coculture process. **B** Exosomes labeled with DiO and taken up by DLBCL cells were observed under a fluorescence microscope (× 400). Slight green fluorescence, indicating exosome uptake by DLBCL cells, could be seen under a confocal fluorescence microscope. **C**–**D** Apoptosis and proliferation of DLBCL cells treated with hBMSC-derived exosomes containing miR-124-3p. The apoptosis rates were increased, and the proliferation of DLBCL cells was inhibited after hBMSC-derived exosomes containing miR-124-3p were added. **E** Protein expression of NFATc1 and cMYC in DLBCL cells determined by western blot analysis. The protein expression levels of NFATc1 and cMYC were suppressed in DLBCL cells cocultured with hBMSC-derived exosomal miR-124-3p. Data are presented as the means ± SD of three independent experiments (*P < 0.05; *** P < 0.001)

Next, to observe whether exosomes derived from hBMSCs can be taken up by DLBCL cells, we cocultured hBMSC-isolated exosomes labeled with DiO with DLBCL cells (Fig. 5A). As shown in Fig. 5B, slight green fluorescence, indicating exosome uptake by DLBCL cells, could be observed under a confocal fluorescence microscope. To determine whether exosomal miR-124-3p from hBMSCs could modulate the biological function of DLBCL cells, proliferation and apoptosis experiments were conducted. The results showed that the apoptosis rates were increased (Fig. 5C) and the proliferation of DLBCL cells was inhibited (Fig. 5D) after hBMSC-derived exosomes containing miR-124-3p were added. Western blot analysis suggested that the protein expression of NFATc1 and cMYC was suppressed in DLBCL cells cocultured with hBMSC-derived exosomal miR-124-3p (Fig. 5E).

**Fig. 6:**
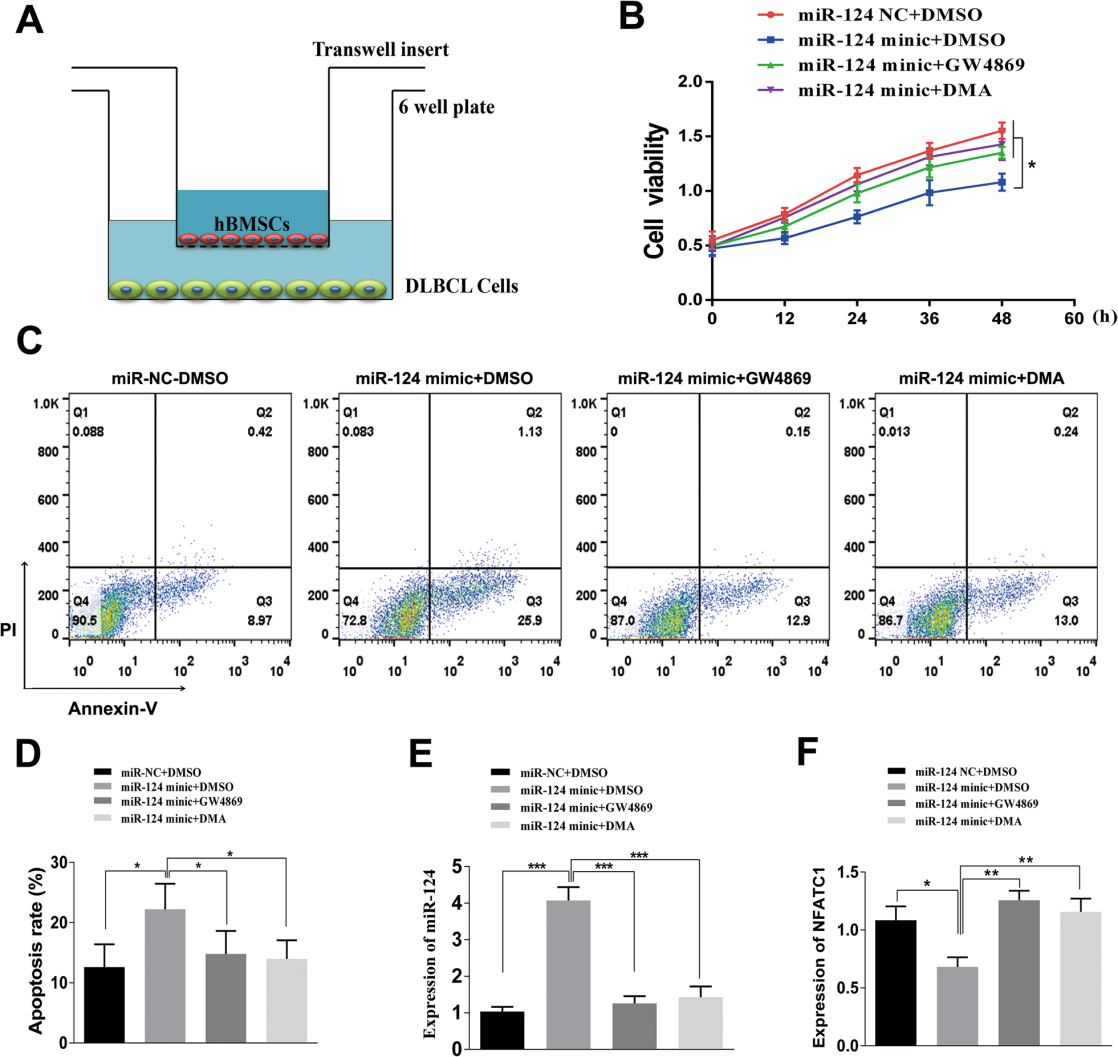
GW4869 and DMA suppress the production of exosomes from hBMSCs, thus reducing the amount of exosomal miR-124-3p and influencing their biological functions. **A** Schematic diagram of the coculture process. **B**–**D** The effects of miR-124-3p derived from hBMSC exosomes on cell proliferation and apoptosis could be rescued by GW4869 or DMA. **E**–**F** GW4869 and DMA treatment resulted in a significant decrease in miR-124-3p levels and an increase in NFATc1 expression, as analyzed by RT-PCR. Data are presented as the means ± SD of three independent experiments (*P < 0.05; **P < 0.01; ***P < 0.001)

To further investigate the effects of hBMSC exosomes on DLBCL cells, miR-124-3p-transfected hBMSCs were directly cocultured with DLBCL cells (Fig. 6A). Furthermore, we employed GW4869 and DMA, two exosome inhibitors, to reduce exosome secretion to determine the function of exosomes in this process. The results demonstrated that miR-124-3p-transfected hBMSCs inhibited proliferation (Fig. 6B) and induced apoptosis (Fig. 6C, D) in DLBCL cells by delivering miR-124-3p via exosomes and that the biological activity of miR-124-3p from hBMSC-derived exosomes could be mitigated by GW4869 or DMA. We also found that coculture with GW4869 and DMA resulted in a significant decrease in miR-124-3p levels (Fig. 6E) and an increase in NFATc1 expression (Fig. 6F), as analyzed by PCR. Thus, GW4869 and DMA could effectively inhibit the production of exosomes from hBMSCs and consequently reduce the transfer of miR-124-3p from hBMSCs to DLBCL cells.

### *MiR-124-3p secreted from hBMSCs inhibits tumor growth *in vivo

**Fig. 7:**
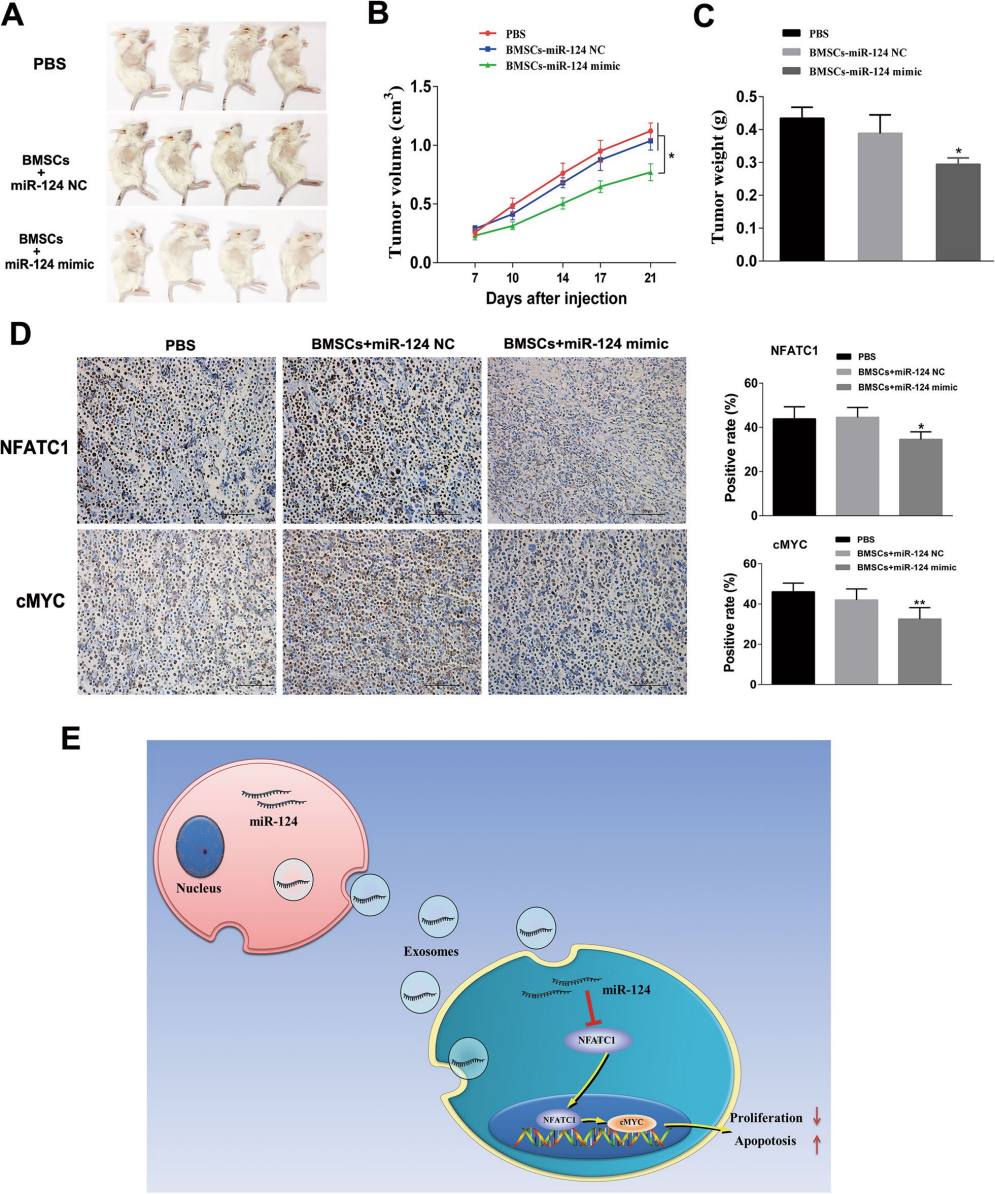
hBMSC overexpressing microRNA-124-3p delays tumor growth in vivo. **A** Representative images of xenograft tumors from the different groups. **B**–**C** Tumor volumes and tumor weights. The tumor volumes and tumor weights in the hBMSC-transfected miR-124-3p group were significantly lower than those in the hBMSC-transfected miR-NC and PBS groups. **D** Expression of NFATc1 and cMYC in xenograft tumors as determined by immunohistochemical staining (× 200). Data are presented as the means ± SD (*P < 0.05; **P < 0.01). **E** Diagram of the regulatory mechanism of hBMSC-derived exosomal miR-124-3p in DLBCL cells. hBMSC-derived exosomal miR-124-3p decreases the expression of cMYC by downregulating NFATc1, thus inhibiting proliferation and enhancing apoptosis

To further evaluate the effect of hBMSCs transfected with miR-124-3p on DLBCL tumor growth in vivo, tumor-bearing mice were injected via the tail vein with hBMSCs transfected with miR-124-3p, hBMSCs transfected with miR-NC or PBS. The expression of miR-124-3p in xenograft tumors in the hBMSC-transfected miR-124-3p group was significantly higher than those in the hBMSC-transfected miR-NC.and PBS groups (Additional file 5: Fig. S4). Tumor volume (Fig. 7A, B) and tumor weight (Fig. 7C) were measured. The tumor volumes and tumor weights in the hBMSC-transfected miR-124-3p group were significantly lower than those in the hBMSC-transfected miR-124-3p NC and PBS groups. These findings support the inhibitory effect of hBMSCs with miR-124-3p on tumor growth in vivo. The immunohistochemical staining results showed that tumors from the hBMSC-transfected miR-124-3p mice showed a lower NFATc1 level (*P* < 0.05) (Fig. 7D) than those from the hBMSC-transfected miR-NC mice and PBS-treated mice.

## Discussion

DLBCL is one of the most aggressive lymphoid malignancies in humans. Relapse or refractory DLBCL patients usually have a median survival of less than 2 years [21]. Human bone marrow-derived mesenchymal stem cells (hBMSCs) can be induced to differentiate into multiple types of mesenchymal tissues and then expanded and genetically modified in vitro, representing therapeutic potential for cancer patients [22]. hBMSCs have been reported to suppress lymphoma cell proliferation in vitro. Intravenous injection of hBMSCs into BALB/c mice with B cell lymphoma showed a reduction in the incidence of lymphoma and improved survival rates [23]. In another study, intraperitoneal injection of hBMSCs into tumor-bearing mice resulted in large stromal infiltration and extensive intratumor necrosis [24]. However, the reported antitumor effects of hBMSCs are still controversial; some evidence indicates that hBMSCs act as proliferative factors in lymphoma growth. hBMSCs can migrate to tumor sites and accelerate the growth of DLBCL cells by protecting them from spontaneous and drug-induced apoptosis [25]. Zhong et al. observed that hBMSCs promote the growth and drug resistance of DLBCL by elevating IL-17A levels and secreting IL-6 [26]. As hBMSCs are a heterogeneous population of activated fibroblasts, they may have distinct effects on the development of different types or stages of non-Hodgkin's lymphoma (NHL) in different microenvironments [27, 28].

Our results indicate that miR-124-3p from hBMSCs suppresses tumor growth both in vitro and in vivo via exosomes.

Exosomes released from hBMSCs carrying miRNAs can mediate communication, which affects cancer cell proliferation, differentiation, and apoptosis [29–31]. For instance, miR-205 from hBMSC-derived exosomes slows prostate cancer progression by inhibiting RHPN2 [32]. Exosomal miR-155-5p secreted by melanoma cells can induce the proangiogenic switch of cancer-associated fibroblasts (CAFs) via the SOCS1/JAK2/STAT3 pathway [33]. MiR-34a in hBMSC-derived exosomes inhibits glioblastoma cell proliferation, migration, invasion and tumorigenesis in vitro and in vivo and promotes chemosensitivity to temozolomide by suppressing MYCN [34]. Our results showed that exosomes containing miR-124-3p could be taken up by DLBCL cells and significantly inhibit the DLBCL proliferation and promote apoptosis.

By analysis of GEO datasets, we found that miR-124-3p was expressed at low levels in DLBCL patients and was associated with a good prognosis, which was confirmed by subsequent experiments. MiR-124-3p is a tumor suppressor miRNA in various hematological tumor types, including multiple myeloma (MM), acute lymphoblastic leukemia (ALL), acute myeloid leukemia (AML), chronic myeloid leukemia (CML), and other B cell lymphomas [35]. The chromosome where miR-124-3p is located is frequently hypermethylated in NHL, with a heterochromatic histone configuration. Hypermethylation results in the inactivation of miR-124-3p and the activation of downstream oncogenes, leading to tumorigenesis [36]. It has been reported that miR-124-3p inhibits NHL cell proliferation and promotes apoptosis via suppression of MYC and BCL2 expression by directly targeting NF-κB p65 [37]. In ovarian cancer, miR-124-3p secreted by ovarian surface epithelial cells can be transferred via exosomes to cancer-associated fibroblasts and inhibit the transition from normal fibroblasts to cancer-associated fibroblasts by targeting sphingosine kinase 1 (SPHK1) [38].

To further investigate the signaling pathways of miR-124-3p in DLBCL cells, we performed bioinformatics analysis and a dual luciferase reporter gene assay. It was suggested that miR-124-3p can target and negatively regulate the expression of the transcription factor NFATc1. In human acute myelogenous leukemia, high NFATc1 expression is associated with poor prognosis [39]. However, in hepatic cancer, ectopic expression of NFATc1 inhibits hepatic cancer proliferation and is associated with good OS [40]. In our clinical data analysis, increased NFATc1 expression was correlated with advanced tumor stage, a high IPI score and poor prognosis in DLBCL patients. Furthermore, knockdown of NFATc1 suppressed the proliferation of DLBCL cells and promoted their apoptosis, and these influences could be partially neutralized by miR-124-3p. We previously demonstrated that NFATc1 can directly upregulate cMYC gene expression by binding to the cMYC promoter; this upregulation is closely related to the growth and survival of tumor cells [41].

## Conclusion

In summary, we found that miR-124-3p derived from hBMSCs could be absorbed by DLBCL cells and inhibit tumor growth both in vivo and in vitro via downregulation of the NFATc1/cMYC pathway (Fig. 7E). These findings likely highlight the potential of exosomal miR-124-3p as a novel molecular target for DLBCL treatment. In the future, many explorations are needed to improve the ability of exosomes to target tumors and to translate this model into clinical application.

## Supplementary Information

**Additional file 1: Table S1.** Primer sequences for RT-qPCR.

**Additional file 2: Figure S1.** Bioinformatics analysis predicted NFATc1 as a target of miR-124-3p. **A** Total of 1027 genes were identified as miR-124-3p-related genes based on analysis with three miRNA-mRNA relation prediction databases. **B** The 1027 miR-124-3p-related genes intersected with the differentially expressed genesand prognostic genesin DLBCL. We obtained 237 miR-124-3p-targeted genes in DLBCL. **C** GO functional enrichment analysis of the 237 miR-124-3p-targeted genes. The results revealed that biological regulation, metabolic process, and response to stimulus were the main biological processes. These genes are related to various cellular components, including the membrane, nucleus, and membrane-enclosed lumen. Concerning molecular function, the major activities of these genes include protein binding, ion binding, and nucleic acid binding. **D** The 237 miR-124-3p-related genes are presented. **E** Protein-protein interactionnetwork analysis showed that NFATc1 was one of the hub genes that exhibited the greatest number of interactions based on analysis with the STRING database and cytoHubba software. **F** Kyoto Encyclopedia of Genes and Genomespathway analysis suggested that the 237 miR-124-3p-related genes were mainly concentrated in the signaling pathways “blood vessel development,” “regulation of cell adhesion” and “positive regulation of cell cycle.”

**Additional file 3: Figure S2.** The significance of miR-124-3p and NFATc1 in cell proliferation/apoptosis by overexpressing NFATc1 with wild-type and mutated 3′-UTR +/− miR-124-3p. **A** The survival rate of cells decreased significantly in the WT-NFATc1+miR-124-3p group compared with the WT-NFATc1+miR-124-NC group and increased in the MUT-NFATc1+miR-124-3p group. **B** In the apoptosis experiment, the apoptosis rate of cells increased in the miR-124-3p mimic groups. The apoptosis rate increased significantly when the 3′-UTR of NFATc1 was mutated.

**Additional file 4: Figure S3.** Isolation and identification of hBMSCs and exosomes. **A** The morphology of hBMSCs. A relatively large number of purified cells with a shuttle shape and swirling arrangement were observed. **B**, **C** The ability of the isolated cells to undergo adipogenic and osteogenic differentiation. A large number of lipid droplets appeared in the cells, and oil red O staining confirmed that the cells underwent adipogenic differentiation. Many red calcium nodules were observed at the cell center with alizarin red staining. **D** Antibodies against CD105, CD90, CD31, CD34, CD45, CD166, CD29, CD11b, HLA-DR, and CD73 were used to identify surface antigens by flow cytometry. CD29, CD90, CD105, CD166, and CD73 were positively expressed, while CD34, CD31, CD45, CD11b, and HLA-DR were negatively expressed. **E** Transmission electron microscopywas used to identify BMSC-derived exosomes. The exosomes were globular or oval in shape and presented a complete lipid membrane. **F** Nanoparticle tracking analysis. The Zeta View nanoparticle tracking analyzer revealed that the majority of exosome particles were approximately 100 nm in size. **G** western blot analysis. The exosome surface marker proteins CD63, TSG 101, and Hsp70 were expressed in BMSC-derived exosomes, whereas calnexin was not. H The miR-124-3p level in miR-124-3p-transfected hBMSCs and exosomes derived from miR-124-3p mimic-treated hBMSCs was significantly higher than that in the respective control cells. The data are expressed as the means ± SD.

**Additional file 5: Figure S4.** The expression of miR-124-3p in xenograft tumors. The miR-124-3p in the hBMSC-transfected miR-124-3p group was significantly higher than those in the hBMSC-transfected miR-NC and PBS groups.

## Data Availability

All data generated and/or analyzed during this study are available from the corresponding author upon reasonable request. The DLBCL-related miRNA expression profiles were retrieved from the Gene Expression Omnibus (GEO) database (https://www.ncbi.nlm.nih.gov/geo/). The GEO Series accession number are GSE29493, GSE40239 and GSE10846.

## Abbreviations

DLBCL: Diffuse large B cell lymphoma
NFATc1: Nuclear factor of activated T cells c1
KEGG: Kyoto Encyclopedia of Genes and Genomes
TEM: Transmission electron microscope
MM: Multiple myeloma
AML: Acute myeloid leukemia
ALL: Acute lymphoblastic leukemia
CML: Chronic myeloid leukemia
3’UTR: 3’Untranslated region
ANOVA: Analysis of variance
BCA: Bicinchoninic acid
BMSCs: Bone marrow-derived mesenchymal stem cells
DAPI: 4,6-Diamidino-2-phenylindole
DEGs: Differentially expressed genes
DEPC: Diethylpyrocarbonate
DMEM: Dulbecco’s modified Eagle’s medium
EDTA: Ethylenediaminetetraacetic acid
FBS: Fetal bovine serum
FITC: Fluorescein isothiocyanate
GAPDH: Glyceraldehyde-3-phosphate dehydrogenase
GEO: Gene Expression Omnibus
hBMSCs: Human bone marrow mesenchymal stem cells
hMSCs: Human mesenchymal stem cells
miRNAs: MicroRNAs
MSCs: Mesenchymal stem cells
MUT: Mutant
NC: Negative control
sh: Short hairpin RNA
PBS: Phosphate-buffered saline
PBST: PBS containing 0.1% Tween-20
PI: Propidium iodide
PVDF: Polyvinylidene fluor
RT-qPCR: Reverse transcription quantitative polymerase chain reaction
SDS-PAGE: Sodium dodecyl sulfate polyacrylamide gelelectrophoresis
TCGA: The Cancer Genome Atlas
WT: Wild type
