# Supplementary material for: Human bone marrow-derived mesenchymal stem overexpressing microRNA-124-3p inhibit DLBCL progression by downregulating the NFATc1/cMYC pathway
Source: Stem Cell Res Ther. 2023 May 29;14:148. doi: 10.1186/s13287-023-03373-w (PMC10228039; doi:10.1186/s13287-023-03373-w)
Supplement: Supplementary file 1 — Additional file 1: Table S1. Primer sequences for RT-qPCR. [file 13287_2023_3373_MOESM1_ESM.docx]

**Table S1.** Primer sequences for RT-qPCR

| Gene | Primer sequence (5′-3′) |
| --- | --- |
| miR-124 | F: 5′- AGATCTCAAAGAGCCTTTGGAAGACG-3′ |
|  | R: 5′-GAATTCTTGCATCTCTAAGCCCCTGT-3′ |
| NFATc1 | F: 5′-AGACTGTGTGGAAATGTAGAGT-3′ |
|  | R: 5′-GTCCCAGAAGGAAAAAGTTTAT-3′ |
| cMYC | F: 5′-ACCAACAGGAACTATGACCTC-3′ |
|  | R: 5′-AAGGCAGTAGCGACCGCAAC-3′ |
| U6 | F: 5′-TCTTTGGAATTCAAGGTCGGGCAGGAAGAGGGCCTA-3′ |
|  | R: 5′-CGCGGATCCTAGTATATGTGCTGCCGAAGC-3′ |
| GAPDH | F: 5′-ATGGAGAAGGCTGGGGCTC-3′ |
|  | R: 5′-AAGTTGTCATGGATGACCTTG-3′ |

RT-qPCR: Reverse transcription quantitative polymerase chain reaction; miR-124: MicroRNA-124; NFATc1: Nuclear factor of activated T cells c1; GAPDH: Glyceraldehyde-3-phosphate dehydrogenase; F: forward; R: reverse
