# Supplementary figures and images for: Human bone marrow-derived mesenchymal stem overexpressing microRNA-124-3p inhibit DLBCL progression by downregulating the NFATc1/cMYC pathway
Source: Stem Cell Res Ther. 2023 May 29;14:148. doi: 10.1186/s13287-023-03373-w (PMC10228039; doi:10.1186/s13287-023-03373-w)

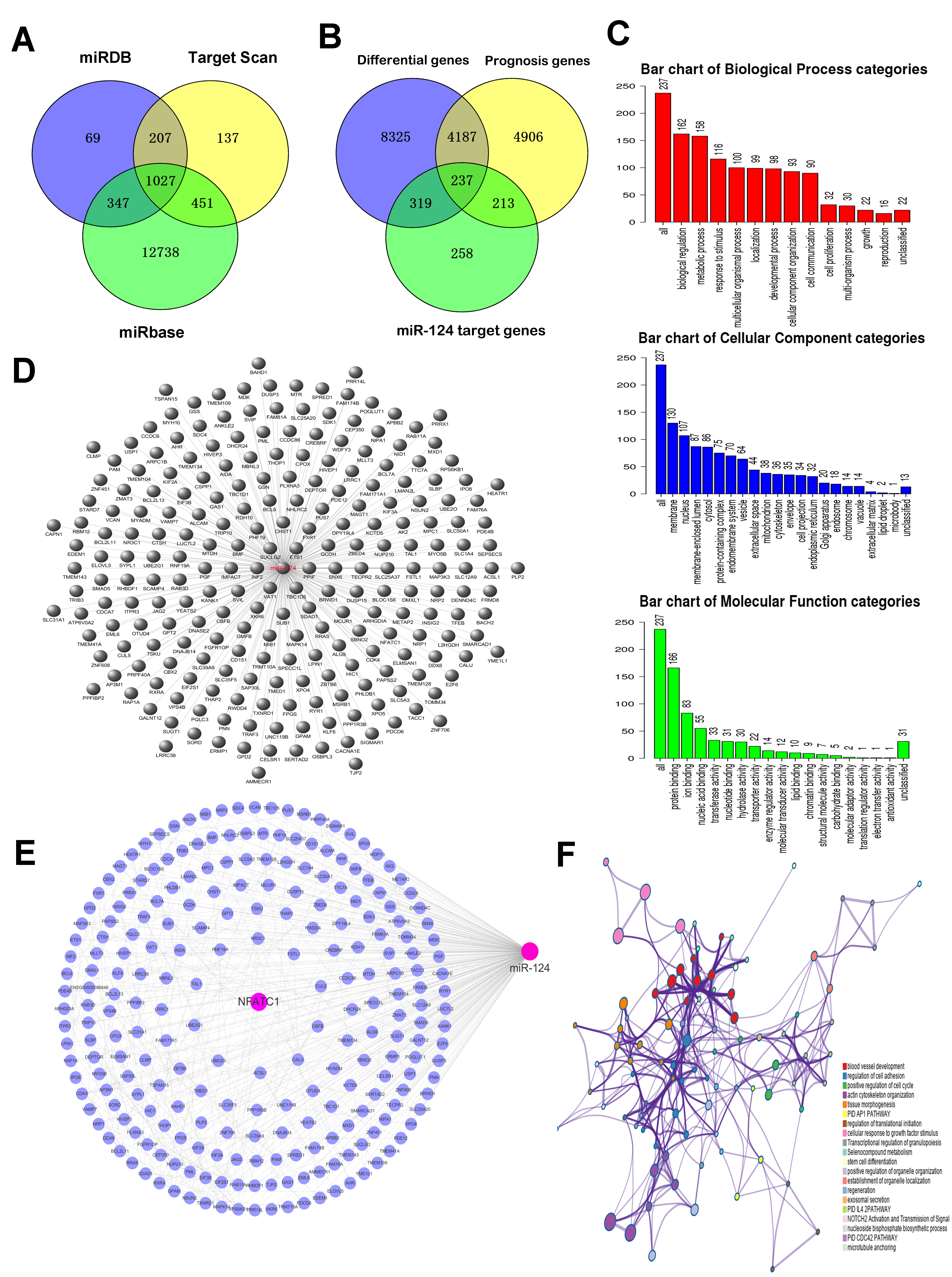

Supplement: Supplementary file 2 — Additional file 2: Figure S1. Bioinformatics analysis predicted NFATc1 as a target of miR-124-3p. A Total of 1027 genes were identified as miR-124-3p-related genes based on analysis with three miRNA-mRNA relation prediction databases. B The 1027 miR-124-3p-related genes intersected with the differentially expressed genesand prognostic genesin DLBCL. We obtained 237 miR-124-3p-targeted genes in DLBCL. C GO functional enrichment analysis of the 237 miR-124-3p-targeted genes. The results revealed that biological regulation, metabolic process, and response to stimulus were the main biological processes. These genes are related to various cellular components, including the membrane, nucleus, and membrane-enclosed lumen. Concerning molecular function, the major activities of these genes include protein binding, ion binding, and nucleic acid binding. D The 237 miR-124-3p-related genes are presented. E Protein-protein interactionnetwork analysis showed that NFATc1 was one of the hub genes that exhibited the greatest number of interactions based on analysis with the STRING database and cytoHubba software. F Kyoto Encyclopedia of Genes and Genomespathway analysis suggested that the 237 miR-124-3p-related genes were mainly concentrated in the signaling pathways “blood vessel development,” “regulation of cell adhesion” and “positive regulation of cell cycle.” [file 13287_2023_3373_MOESM2_ESM.tif]

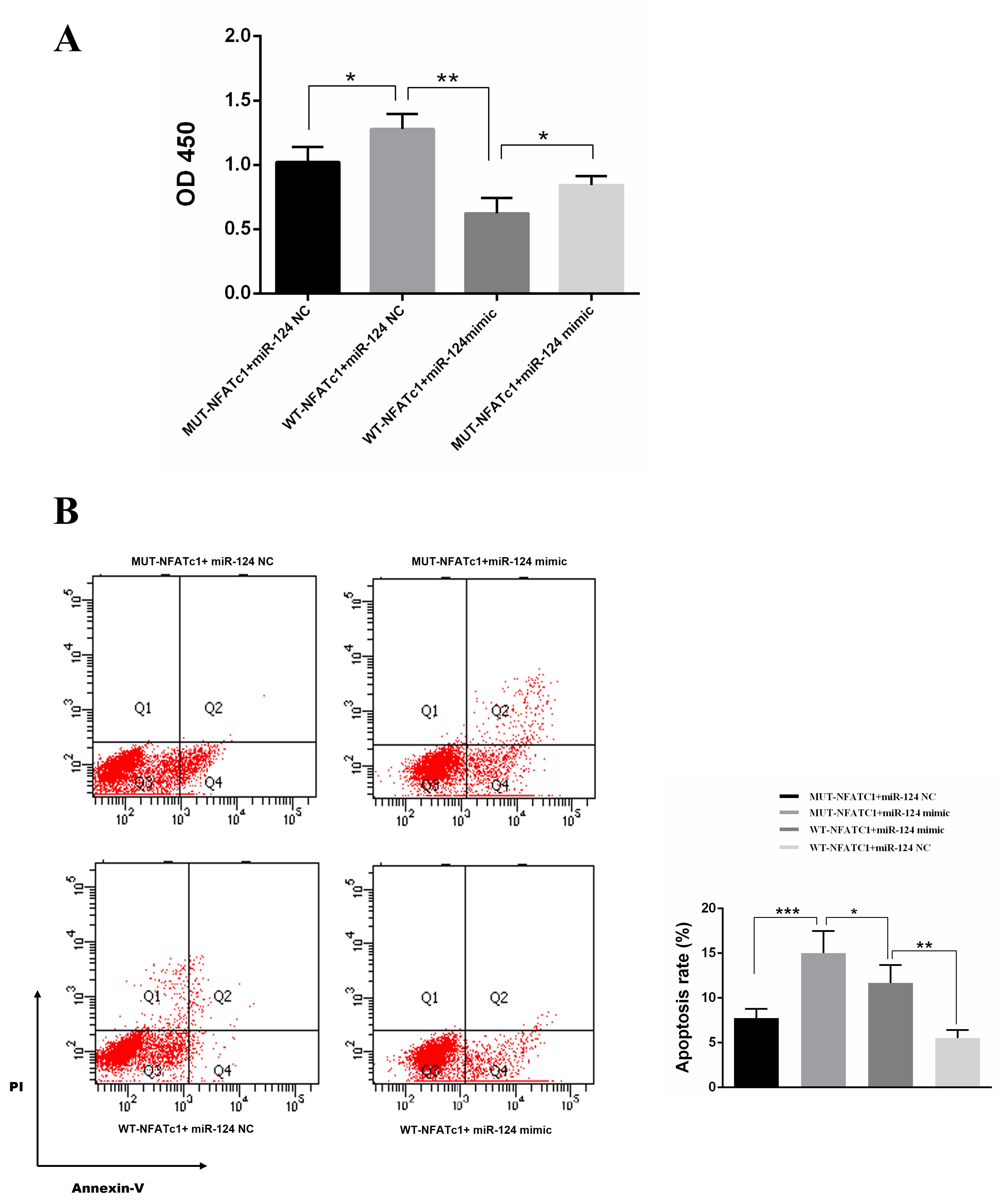

Supplement: Supplementary file 3 — Additional file 3: Figure S2. The significance of miR-124-3p and NFATc1 in cell proliferation/apoptosis by overexpressing NFATc1 with wild-type and mutated 3′-UTR +/− miR-124-3p. A The survival rate of cells decreased significantly in the WT-NFATc1+miR-124-3p group compared with the WT-NFATc1+miR-124-NC group and increased in the MUT-NFATc1+miR-124-3p group. B In the apoptosis experiment, the apoptosis rate of cells increased in the miR-124-3p mimic groups. The apoptosis rate increased significantly when the 3′-UTR of NFATc1 was mutated. [file 13287_2023_3373_MOESM3_ESM.jpg]

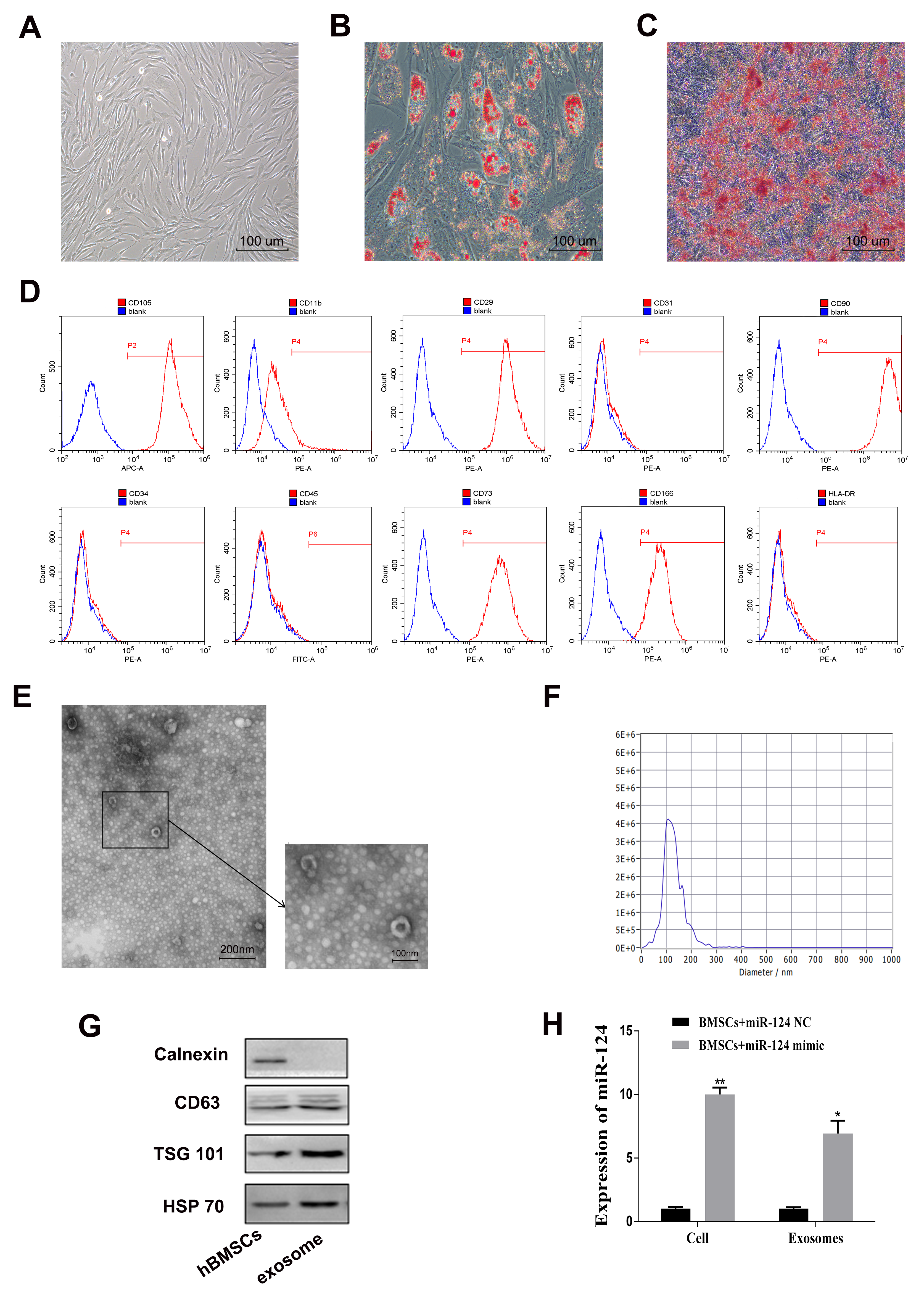

Supplement: Supplementary file 4 — Additional file 4: Figure S3. Isolation and identification of hBMSCs and exosomes. A The morphology of hBMSCs. A relatively large number of purified cells with a shuttle shape and swirling arrangement were observed. B, C The ability of the isolated cells to undergo adipogenic and osteogenic differentiation. A large number of lipid droplets appeared in the cells, and oil red O staining confirmed that the cells underwent adipogenic differentiation. Many red calcium nodules were observed at the cell center with alizarin red staining. D Antibodies against CD105, CD90, CD31, CD34, CD45, CD166, CD29, CD11b, HLA-DR, and CD73 were used to identify surface antigens by flow cytometry. CD29, CD90, CD105, CD166, and CD73 were positively expressed, while CD34, CD31, CD45, CD11b, and HLA-DR were negatively expressed. E Transmission electron microscopywas used to identify BMSC-derived exosomes. The exosomes were globular or oval in shape and presented a complete lipid membrane. F Nanoparticle tracking analysis. The Zeta View nanoparticle tracking analyzer revealed that the majority of exosome particles were approximately 100 nm in size. G western blot analysis. The exosome surface marker proteins CD63, TSG 101, and Hsp70 were expressed in BMSC-derived exosomes, whereas calnexin was not. H The miR-124-3p level in miR-124-3p-transfected hBMSCs and exosomes derived from miR-124-3p mimic-treated hBMSCs was significantly higher than that in the respective control cells. The data are expressed as the means ± SD. [file 13287_2023_3373_MOESM4_ESM.tif]

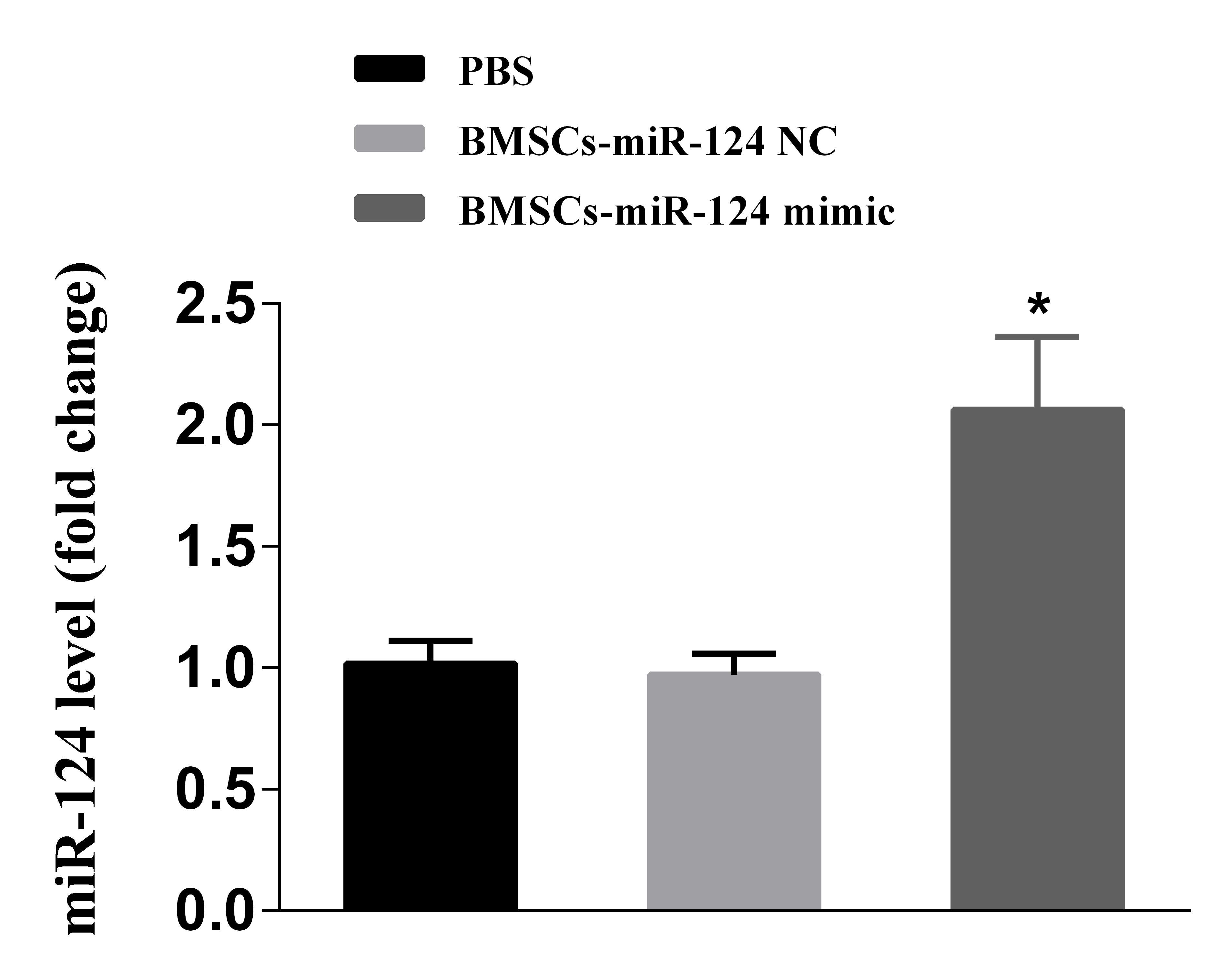

Supplement: Supplementary file 5 — Additional file 5: Figure S4. The expression of miR-124-3p in xenograft tumors. The miR-124-3p in the hBMSC-transfected miR-124-3p group was significantly higher than those in the hBMSC-transfected miR-NC and PBS groups. [file 13287_2023_3373_MOESM5_ESM.tif]
